# Cytokine IL-5 and HGF: combined prediction of non-/low immune response to hepatitis B vaccination at birth in infants born to HBsAg-positive mothers

**DOI:** 10.3389/fcimb.2024.1332666

**Published:** 2024-03-01

**Authors:** Guanyong Ou, Ling Qing, Li Zhang, Yang Yang, Guoguo Ye, Ling Peng, Yanjie Li, Liuqing Yang, Yingxia Liu

**Affiliations:** ^1^ National Clinical Research Center for Infectious Disease, State Key Discipline of Infectious Disease, The Third People’s Hospital of Shenzhen, Second Hospital Affiliated to Southern University of Science and Technology, Shenzhen, China; ^2^ School of Medicine, Southern University of Science and Technology, Shenzhen, China; ^3^ Graduate Collaborative Training Base of Shenzhen Third People’s Hospital, Hengyang Medical School, University of South China, Hengyang, Hunan, China

**Keywords:** hepatitis B vaccine, immune response, infant, cytokine, IL-5, HGF, non/low response

## Abstract

**Background:**

The immune response to hepatitis B vaccine may be influenced by numerous factors, and patients with non/low response re-exposed to hepatitis B virus remain susceptible. Thus, a better understanding of the underlying mechanisms of non/low immune response in infants born to Hepatitis B surface antigen (HBsAg)-positive mothers is essential.

**Methods:**

100 infants born to HBsAg-positive mothers from 2015 to 2020 were enrolled in the study, further divided into the non/low response group (n=13) and the moderate strong response group (n=87) based on the quantification of hepatitis B surface antibody at 12 months of age. The differential expression of 48 immune-related cytokines in the two groups was compared and analyzed in detail. The key cytokines were further identified and clinically predictive models were developed.

**Results:**

We found that 13 cytokines were lowly expressed and one cytokine was highly expressed in the non/low response group, compared with the moderate strong response group at birth. In addition, 9 cytokines were lowly expressed and one cytokine was highly expressed in the non/low response group at 12 months of age. Furthermore, we found that IL-5 and HGF were promising predictors for predicting the immunization response to hepatitis B vaccine in infants, and the combination of the two cytokines showed the best predictive efficiency, with an area under the curve (AUC) value of 0.844.

**Conclusion:**

The present study provides a theoretical basis on cytokines for developing and implementing effective immunotherapies against non/low immune response in infants born to HBsAg-positive mothers.

## Introduction

Chronic Hepatitis B (CHB) is a serious global public health problem. According to the World Health Organization (WHO), an estimated 257 million people worldwide are infected with the hepatitis B virus (HBV). Additionally, approximately 887,000 people die each year from diseases related to HBV infection, including 30% from cirrhosis and 45% from primary hepatocellular carcinoma (Bertoletti and Ferrari, 2016; Revill et al., 2019).

Receiving the hepatitis B vaccine is the most effective strategy for preventing HBV infection (Locarnini et al., 2015). To provide maximal protection against mother-to-child transmission, it is advised that newborns are immunized with this vaccine immediately after birth (<24 hours), followed by doses at one and six months of age. Subsequent to the completion of the vaccination series, testing for serological markers of hepatitis B is recommended (Pattyn et al., 2021; Lanini et al., 2019). Research has identified that titers of Hepatitis B surface antibody (anti-HBs) below 10 mIU/mL pose a risk factor for the reactivation of the virus (Yi et al., 2016; Wu et al., 2018), whereas levels equal to or greater than 10 mIU/mL offer immunoprotection, especially critical for infants born to Hepatitis B surface antigen (HBsAg)-positive mothers (Nishida et al., 2018). The implementation of hepatitis B vaccination protocols has notably decreased the incidence of HBsAg infections. The progression and outcome of an HBV infection are significantly determined by the interplay between the virus and its host, with the age at infection being a crucial determinant of the disease’s chronicity (Iannacone and Guidotti, 2022; Kwan et al., 2021). Infants and neonates under one year face a 90% risk of developing a chronic condition following exposure (Fisman et al., 2002; Zimmermann and Curtis, 2019). Further studies have highlighted individuals living with HBsAg-positive family members are at an elevated risk, particularly when those who are non-responders or have low response rates encounter infected populations, thereby increasing their susceptibility to HBV (Zhou et al., 2017; Shahmoradi et al., 2012). This situation underscores the ongoing challenges in curtailing the spread of HBV infections.

The immune response elicited by hepatitis B vaccination is a complex process influenced by an array of factors, including characteristics of the vaccine itself, host organismal factors, and viral attributes (Zimmermann and Curtis, 2019). Studies have documented that the production of anti-HBs by B lymphocytes was dependent on the stimulation of multiple signals (Ma et al., 2019; Le Bert et al., 2020). Upon administration of the hepatitis B vaccine, antigen-presenting cells (APCs), such as dendritic cells and macrophages, are activated (Horst et al., 2021). These cells then capture the antigen and migrate to proximal lymph nodes where they undergo maturation and process the antigen. During this process, HBsAg is presented to T cells in the context of peptide-MHC class II complexes. This antigen presentation is crucial for the activation of CD4+ T lymphocytes, which, depending on the cytokine milieu, can differentiate into either Th1 or Th2 cells. Th2 cells, upon activation, promote the proliferation of themselves and secrete cytokines that are instrumental in activating B cells. These activated B cells then undergo differentiation into plasma cells, which are responsible for the production of anti-HBs antibodies (Khanam et al., 2021; Peeridogaheh et al., 2018). The activation of B cells also involves interactions with co-stimulatory molecules present on the surface of Th cells (Bertoletti and Ferrari, 2016), highlighting the intricate interplay between cellular components of the immune system. Moreover, the hepatitis B vaccine induces the body to produce protective antibodies through a mechanism that necessitates the activation of Th cells and the coordinated secretion of cytokines by Th1 and Th2 cells (Bertoletti and Ferrari, 2016; Gu et al., 2020). The cytokine profiles of Th1 and Th2 cells are distinct and have differing effects on the immune response (Jafarzadeh and Shokri, 2012; Zhu, 2018). Th1 cells are known for their role in promoting cell-mediated immunity, whereas Th2 cells support humoral immunity by stimulating antibody production (Adugna, 2023; Zhang et al., 2014). The balance between Th1 and Th2 responses is critical in determining the efficacy of the vaccine-induced immune response. A predominance of Th1 responses tends to favor a more robust cell-mediated immunity, while a Th2-biased response enhances antibody production (Butcher and Zhu, 2021). The dynamic interplay between Th1 and Th2 cells, characterized by both synergistic and antagonistic interactions, is essential for achieving an optimal immune response to the hepatitis B vaccine. This balance is influenced by various factors, including the cytokine environment, which in turn can be affected by the vaccine, host, and viral factors (Van Eden et al., 2002; Lin et al., 2023). It is evident that cytokines and immune cells play a pivotal role in orchestrating the immune response.

In this study, we conducted a comprehensive investigation into the expression of 48 immune-related cytokines in the peripheral blood plasma of infants born to HBsAg-positive mothers. Our primary objective was to identify specific cytokines that correlate with a non/low response to hepatitis B vaccination among these infants. Building on these findings, we aimed to develop a predictive model for identifying infants at risk of non/low response to hepatitis B vaccination. This model is based on a combination of clinical indicators and cytokine expression profiles. Through our analysis of cytokine expression in infants born to HBsAg-positive mothers, we seek to lay a theoretical foundation for the development and implementation of more effective antiviral immunotherapies.

## Materials and methods

### Study participants and clinical data collection

**Figure 1:**
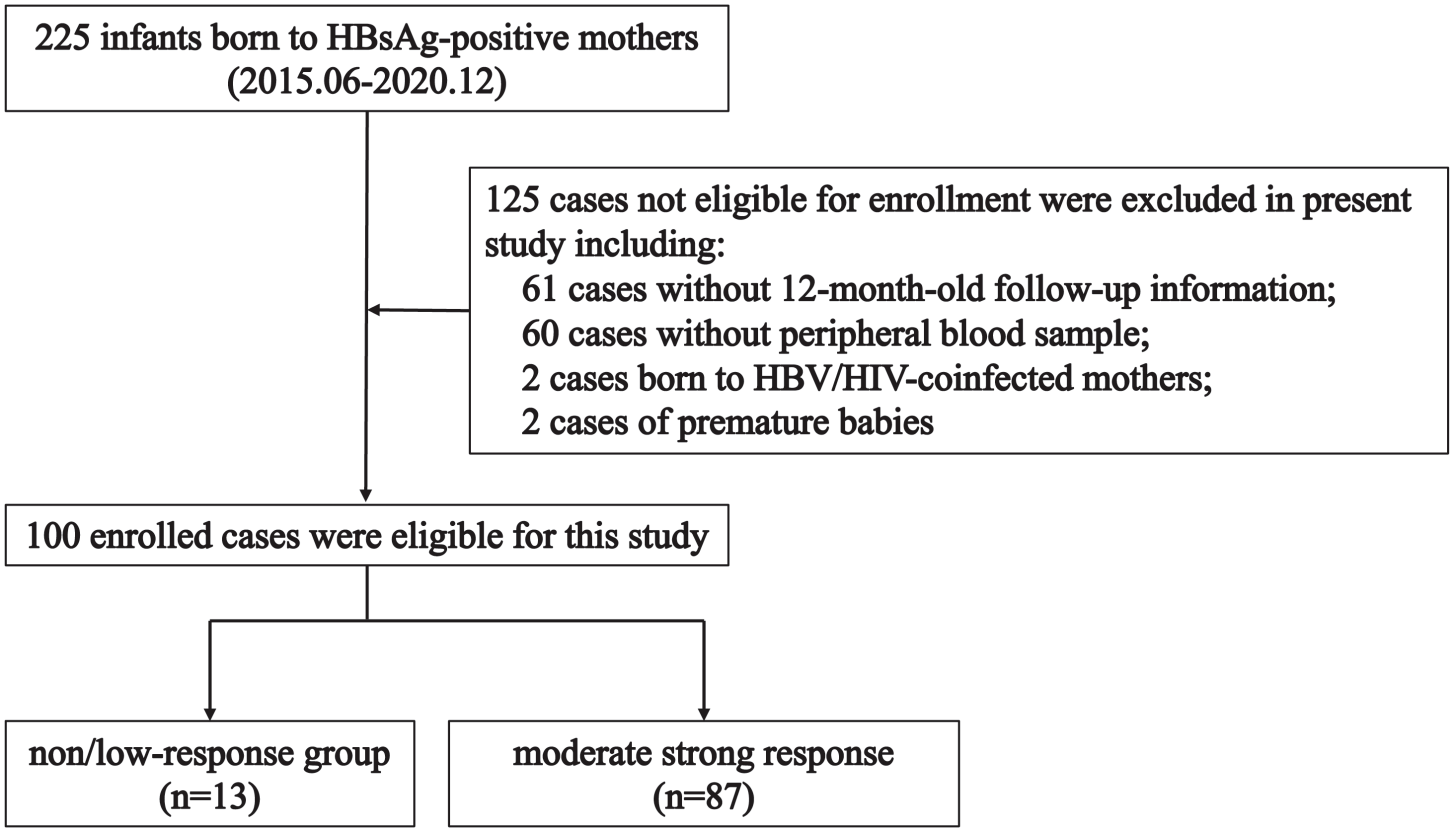
Overall flowchart of study design.

From June 2015 to December 2020, 225 infants born to HBsAg-positive mothers were recruited as study participants from Shenzhen Third People’s Hospital in present study. All newborn infants were vaccinated with Hepatitis B Immunoglobulin (HBIG) and recombinant hepatitis B vaccine at birth, and also received recombinant hepatitis B vaccine at 1 and 6 months of age, respectively. HBsAg-positive mothers were screened for HBV markers and liver function, and the clinical baseline information of mothers was collected and recorded. In addition, 125 infants not eligible for enrollment were excluded in the present study (including 61 cases without 12-month-old follow-up information, 60 cases without peripheral blood sample, 2 cases born to HBV/HIV-coinfected mothers, and 2 cases of premature babies), and100 eligible cases were enrolled in this study (**Figure 1**). Ethical approval of the present study was permitted by the ethics committees of The Third People’s Hospital of Shenzhen (2018-014) and the study was conducted in accordance with the International Conference on Harmonization Guidelines for Good Clinical Practice and the 1975 Declaration of Helsinki and institutional ethics guidelines.

### Evaluation of immunization efficacy against hepatitis B in infants

Referring to the clinical management process for blocking mother-to-child transmission of hepatitis B virus (Hou et al., 2019), this study assessed the efficacy of hepatitis B vaccination in preventing mother-to-child transmission of the hepatitis B virus (HBV). Immunization response was classified based on the presence of hepatitis B surface antibodies (HBsAb) as follows:

(1) No response: Characterized by an HBsAb level below 10 mIU/ml, indicating a lack of protective immunity against HBV.
(2) Low response: Defined by HBsAb levels ranging from 10 to 100 mIU/ml, suggesting a minimal protective effect.
(3) Moderate strong response: Indicated by HBsAb levels of 100 mIU/ml or higher, reflecting adequate immunological protection against HBV.

Infants enrolled in the study were classified into these distinct groups according to their HBsAb response levels. This classification enabled a detailed analysis of the immunization’s effectiveness in generating a protective immune response against hepatitis B among infants born to HBsAg-positive mothers.

### Measure of plasma cytokines

The plasma samples of infants were collected from infants at birth (before active and passive immunization) and 12 months of age (after immunization), and the plasma samples were collected from mothers before delivery. All samples were stored at -80°C in the refrigerator for laboratory testing and analysis. The expression levels of 48 cytokines were measured using Bio-Plex Pro Human Cytokine Screening Panel (Bio-Rad, Berkeley, Calif) as previously reported (Liu et al., 2020). The detailed information of 48 cytokines was shown in **Supplementary Table S1**.

### Least absolute shrinkage and selection operator regression analysis

Least absolute shrinkage and selection operator (LASSO) is a regularization and descending dimension method, which can be used in key cytokines screening combined with receiver operating characteristic (ROC) curve analysis. We first performed a LASSO regression model with 10-fold cross-validation and 1000 bootstrap samples carried out to remove over-fitting regarding cytokines related to immune response for feature selection through R package “Glmnet” (Engebretsen and Bohlin, 2019). Further, ROC curve analysis through R package “pROC” (Robin et al., 2011) was performed to assess the predictive accuracy of identified cytokines. Moreover, the combined values for predicting individual cytokine were assessed using binary logistic regression to predict immune response to hepatitis B immunization.

### Statistical analysis

HBV DNA was log-transformed with a base of 10. Measurement data: Shapiro-Wilk test for normality, Levene test for chi-square test between groups, t-test for comparison between two groups conforming to normal distribution and chi-square; expressed as mean ± standard deviation, non-normal distribution by a non-parametric test, expressed as median (25%, 75% interquartile range). Count data: expressed as frequency or rate (%), χ^2^ test was used for comparison between groups; ROC curve was used and area under the curve (AUC) was assessed to analyze the validity of cytokine indexes to predict immune response to hepatitis B immunization. A statistically significant difference was defined as p<0.05.

## Results

### Clinical characteristics

The enrollment flow of the present study was shown in **Figure 1**. Among 100 HBsAg-positive mothers, the median age of these patients in non/low-response group (n=13) was 30 years, ranging from 27 to 33 years, and the median age of these patients in moderate strong response moderate strong response group (n=87) was 29 years, ranged from 26 to 32 years (**Supplementary Table S2**). All clinical characteristics of HBsAg-positive mothers, including age, gestational weeks, ALT, AST, BUN and especially HBV-DNA and maternal comorbidities, did not manifest significant differences between non/low-response group and moderate strong response group (**Supplementary Table S2**). Next, we further analyzed whether the factors associated with the birth of the infants affected the immune response. We first analyzed the delivery mode of the infants, and no significant differences were observed between the two groups, regardless of whether the delivery mode was natural or cesarean (p=0.074) (**Supplementary Table S3**), and there were no statistically significant differences in the sex or the feeding mode between the two groups (all p>0.05). Further analysis of the effect of birthweight on immunization response revealed that the median weight of infants was 3600 g in the non/low-response group and 3200 g in moderate strong response group, respectively, and the differences were statistically significant (p=0.005) (**Supplementary Table S3**).

Particularly, we meticulously analyzed the correlations between infant birth weight and maternal clinical characteristics to elucidate potential markers or predictors of neonatal health in the context of hepatitis B exposure. Our findings revealed a modestly positive correlation between infant birth weight and four clinical indices: aspartate aminotransferase (AST), total bilirubin (TBIL), Blood loss at delivery (BLD), and gestational week (**Supplementary Figure S1A**). This suggests that higher values in these maternal clinical indices, within physiological limits, may be associated with increased birth weight, potentially indicating a healthier neonatal outcome. Furthermore, our analysis uncovered a notably strong positive correlation between alanine aminotransferase (ALT) and AST (R=0.91, p=7.1e-39), highlighting a significant link between these liver enzymes in HBsAg-positive mothers (**Supplementary Figure S1B**). This relationship underscores the liver’s stressed condition in hepatitis B, as both enzymes are commonly elevated in liver damage or inflammation. The strong correlation suggests that these enzymes could serve as reliable markers for monitoring liver health in HBsAg-positive pregnant women. Additionally, we identified a significant negative correlation between total bilirubin (TBIL) and blood urea nitrogen (BUN) (R=-0.32, p=1.2e-3) (**Supplementary Figure S1C**). This inverse relationship might indicate a complex interplay between liver function (as reflected by bilirubin levels) and renal health (as indicated by BUN levels) in the context of hepatitis B infection. Lower BUN levels associated with higher TBIL could suggest a compensatory renal response to altered liver function.

### Differential expression analysis of cytokine profile in infants in different immunization response groups

**Figure 2:**
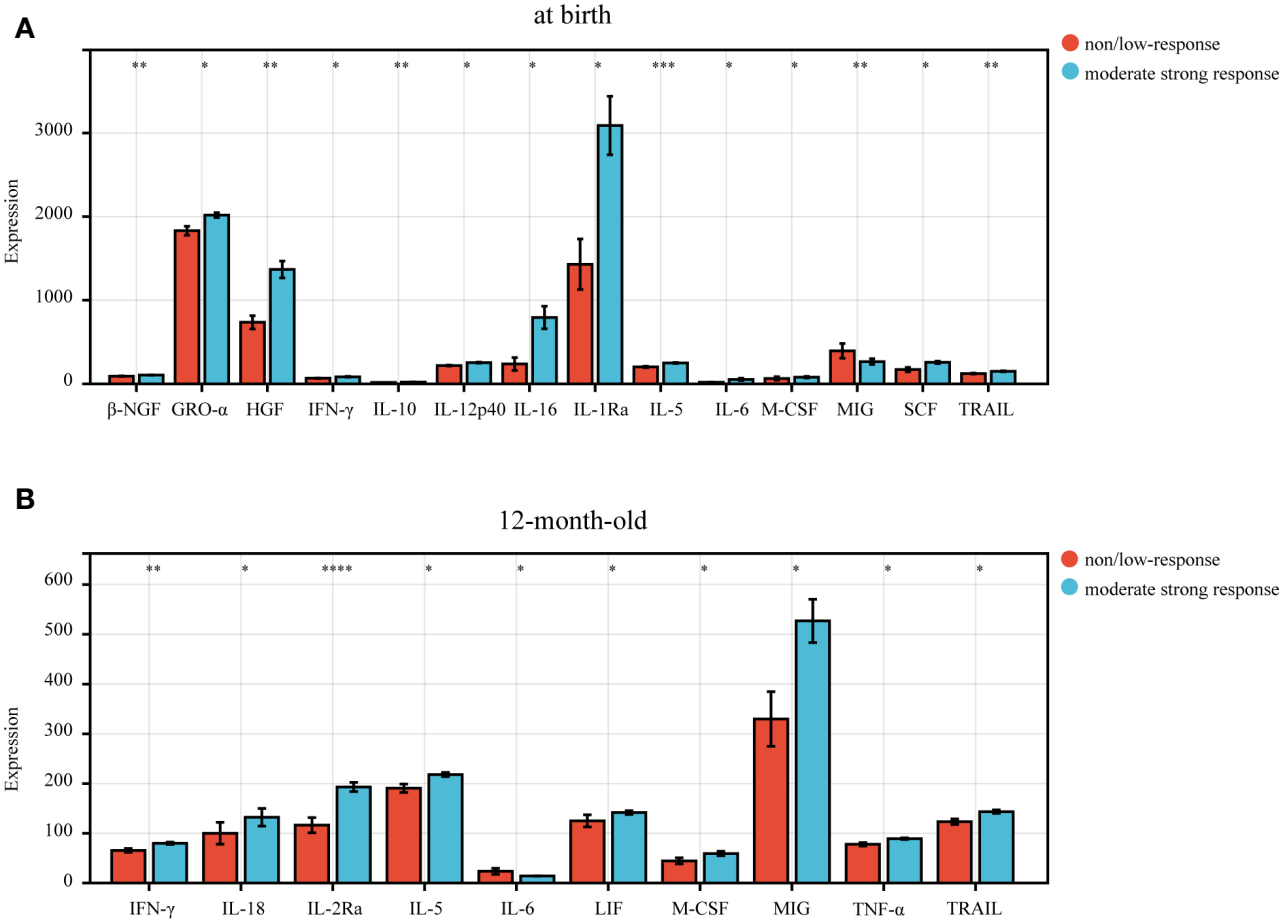
The differential expression level of cytokines in infants born to HBsAg-positive mothers. **(A)** 14 cytokines differentially expressed in the non/low-response and moderate strong response groups in newborn infants at birth. **(B)** 10 cytokines differentially expressed in the non/low-response and moderate strong response groups in 12-month-old infants. P<0.05 indicated statistical significance. P-values were showed as: *, P <0.05; **, P <0.01; ***, P <0.001; ****, P <0.0001.

To further investigate the relationship between immunization response to hepatitis B vaccine and cytokines, we first analyzed the expression levels of 48 plasma cytokines in infants at birth (**Supplementary Figure S2A**). The results showed that 14 of 48 cytokines were differentially expressed in infants at birth (**Figure 2A**). The expression levels of β-NGF (Beta nerve growth factor), GRO-α (Growth regulated oncogene α), HGF (Hepatocyte growth factor), IFN-γ (Interferon gamma), IL-10 (interleukin 10), IL-12p40 (interleukin 12 (p40)), IL-16 (interleukin 16), IL-1Ra (interleukin 1 receptor antagonist), IL-5 (interleukin 5), IL-6 (interleukin 6), M-CSF (Macrophage colony Stimulating Factor 1), SCF (Stem cell factor) and TRAIL (TNF-related apoptosis-inducing ligand), in the non/low response group, were significantly lower than those in the moderate strong response group, and MIG (Mitogen-inducible gene) expression levels were significantly higher than that in the moderate strong respond group at birth (all p < 0.05) (**Figure 2A**). And IL-1Ra expression level was the highest in the moderate strong response group, and GRO-α was the highest in the non/low response group at birth (**Figure 2A**).

We further explored the expression of 48 cytokines in infants at 12 months of age (**Supplementary Figure S2B**) and found that the expression levels of 9 cytokines, including IFN-γ, IL-18 (interleukin 18), IL-2Ra (interleukin 2 receptor antagonist), IL-5, LIF (Leukemia Inhibitory Factor), M-CSF, MIG, TNF-α (tumor necrosis factor alpha), and TRAIL, were significantly lower in the non/low response group than in the moderate strong response group (**Figure 2B**), while the expression level of IL-6 in non/low response group was significantly higher than in moderate strong response group (p<0.05).

**Figure 3:**
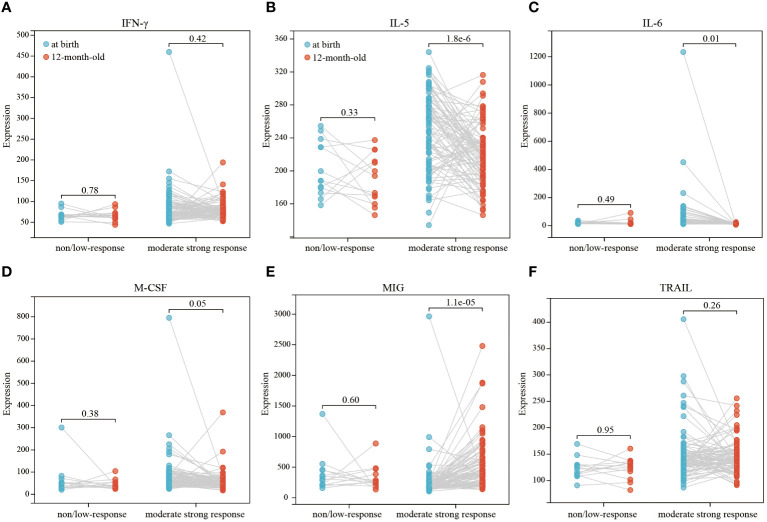
Paired differential expression analysis of 6 cytokines (IFN-γ, IL-5, IL-6, M-CSF, MIG, TRAIL) in different response groups **(A–F)**. Paired‐samples t‐test analysis for at birth/12-month-old pairs was used to depict the differential levels, and P<0.05 indicated statistical significance.

Our comparative analysis highlighted significant differences in the levels of IFN-γ, IL-5, IL-6, M-CSF, MIG, and TRAIL in infants at birth and at 12 months of age, with all differences being statistically significant (p<0.05) (**Figures 2A, B**). Thus, we further conducted a paired differential expression analysis on these six cytokines at birth and at 12 months of age (**Figures 3A-F**). This analysis revealed that, specifically, IL-5 (**Figure 3B**), IL-6 (**Figure 3C**), and MIG (**Figure 3E**) exhibited differential expression in the moderate strong response group. Conversely, we observed no significant differences in the expression of these six cytokines in the non/low response group (**Figure 3**).

### Independent predicative value of cytokines for the immunization response to hepatitis B vaccine in infants

**Figure 4:**
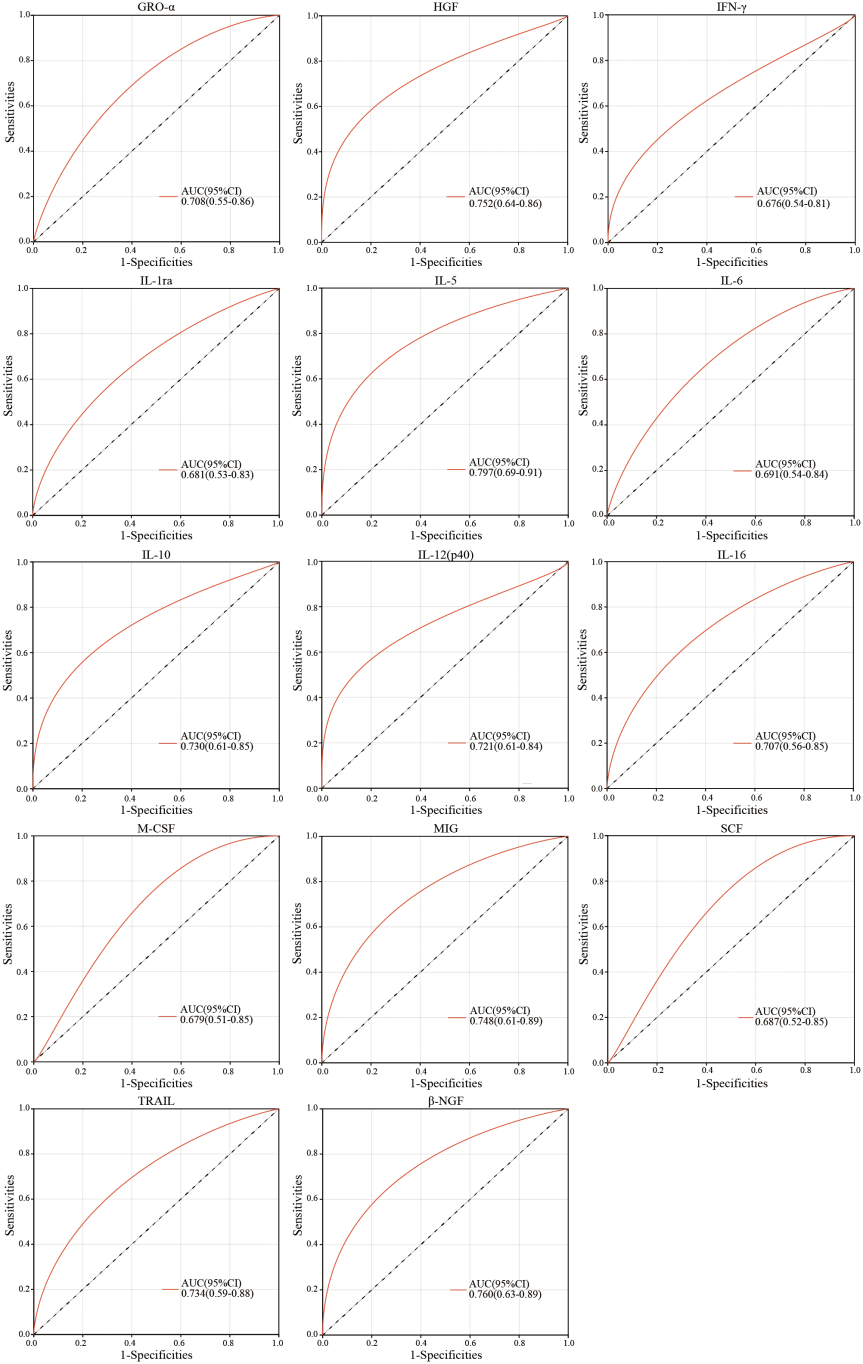
ROC curve analysis of peripheral plasma cytokine levels for infants at birth in the non/low-response and moderate strong response groups.

Furthermore, we performed ROC curve analysis to evaluate the predictive efficiency of single cytokines differentially expressed for immunization response in infants at birth. The results demonstrated that AUC value of ROC curve was 0.797 for IL-5, followed by 0.760 for β-NGF (**Figure 4**), and the AUC of other cytokines ranged from 0.676 to 0.752 (**Figure 4**), suggesting the appreciable reliability of 14 cytokines as predictive indicators for different immunization response to hepatitis B vaccine in infants.

### IL-5 and HGF are key cytokine predictors for predicting the immunization response in infants

**Figure 5:**
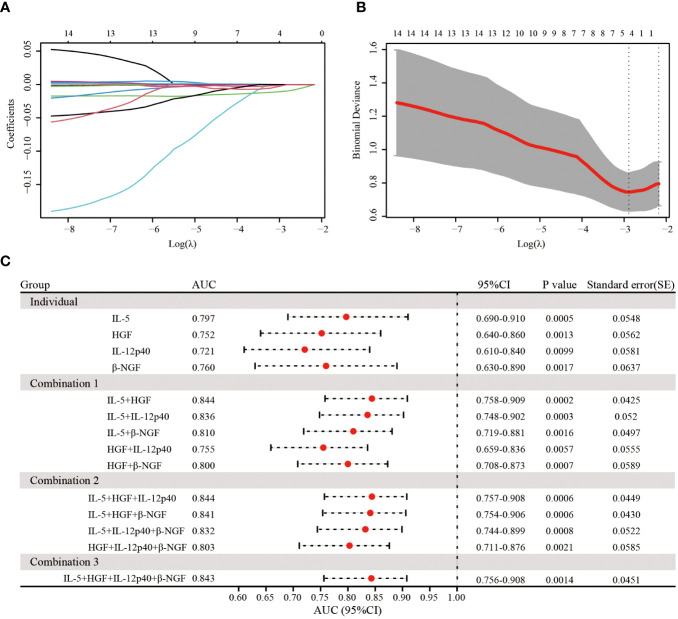
Lasso regression analysis of 14 cytokines and a predictor model construction based on identified 4 cytokines. **(A)** LASSO regression analysis for 14 cytokines. **(B)** The relationship of the most appropriate log (λ) value and binomial deviance in LASSO regression mode. **(C)** The forest plot showed the predictive effect of different combination of 4 identified cytokines for different immune response through ROC curve analysis.

Then, to further identify the key differentially expressed cytokines at birth that were significantly correlated with the immunization response of infants born to HBsAg-positive mothers, we employed LASSO-Logistic regression to screen for potential predictors (**Figures 5A, B**). The results showed that a total of 4 cytokines, including IL-5, HGF, IL-12p40 and β-NGF, were identified as key cytokines. The parameters in LASSO model were shown in **Supplementary Table S4**. As shown in **Supplementary Table S5**, the negative coefficients of 4 identified cytokines, including IL-5, HGF, IL-12p40 and β-NGF, in the LASSO-Logistic regression model, suggested that the four cytokines were protective factors for infants born to HBsAg-positive mothers.

Next, we tested different combinations of 4 cytokines to predict immune responses (**Figure 5C**). The combination of IL-5 and HGF exhibited the highest AUC of 0.844, the same as the combination of IL-5, HGF and IL-12p40, as followed by the combination of 4 cytokines with an AUC of 0.843 (**Figure 5C**). In addition, the combination of IL-5, HGF and β-NGF also manifested a practical predictive and diagnostic value with an AUC of 0.841(**Figure 5C**), and the other combination of 4 cytokines also showed an excellent predictive effect, with the AUC ranging from 0.755 to 0.836 (**Figure 5C**). These results suggest that the 4 identified cytokines can better predict the immune response of infants born to HBsAg-positive mothers after hepatitis B immunization and that the combination of IL-5 and HGF was a valid diagnostic determinant.

## Discussion

Numerous factors may influence the immune response to HBV vaccination (Zimmermann and Curtis, 2019). Nevertheless, the mechanisms of response to hepatitis B vaccine, especially non or low response, remain to be clearly determined. The most common mode of transmission of hepatitis B virus is from mother to child during birth and delivery (Yuen et al., 2018; Yi et al., 2016). Especially when the non/low-response patients are exposed to HBV again, they remain susceptible and may even develop into HBV carriers (Wang et al., 2019). Thus, a better understanding of the underlying mechanism in infants’ non/low immunization response is essential for developing and implementing effective antiviral therapies.

In this study, 100 infants born to HBsAg-positive mothers were enrolled and the relationships between expression levels of plasma cytokines and the immunization response to hepatitis B vaccine were analyzed in detail. The non/low response rate of hepatitis B immunization in infants in this study was 13%, which was similar to the previous study (Jiang et al., 2021), but the non/low response rate in this study was lower than that of other studies in China. The difference in results may be attributable to the hospital and region where this study was conducted, as the hospital is an infectious disease specialist that ensures that all newborns can receive hepatitis B vaccine and hepatitis B immunoglobulin no more than 12 hours after birth. Previous studies have reported that infants fed artificially and whose HBsAg-positive mothers had HBV DNA≥1×10^7^ copies/ml were prone to non/low response to hepatitis B immunization (Hou et al., 2019; Ko et al., 2020). In this study, infant birth weight was found to be associated with the effect of hepatitis B immune response, which is the same as the results of previous studies (Wang et al., 2016). Our study identified a significant negative correlation between neonatal birth weight and the immune response to hepatitis B vaccination. Specifically, infants with a median birth weight of 3200 grams showed a moderate to strong immune response, whereas those weighing 3600 grams were more likely to have a non/low response. This suggests that lower birth weight may be associated with a stronger immune reaction to HBV vaccination. This negative relationship underscores the complexity of neonatal immune system maturation and its interaction with physiological factors such as birth weight. Further research is needed to elucidate the mechanisms underlying this association and to determine whether similar patterns are observed in broader populations.

Recent studies have highlighted IL-10 as a critical cytokine predominantly produced by Th2 cells. It plays a crucial role in immune regulation by modulating the activity of macrophages, T cells, B cells, and NK cells (Sabat et al., 2010). The primary functions of IL-10 include the suppression of pro-inflammatory cytokine production, the limitation and resolution of inflammatory responses, and the facilitation of B cell proliferation and differentiation for antibody production (Sabat et al., 2010; El-Emshaty et al., 2015; Körber et al., 2021). Our findings corroborated these observations, indicating lower plasma expression level of IL-10 in individuals with a non/low response to the hepatitis B vaccine. Furthermore, our study expands the understanding of cytokine involvement by demonstrating that IL-5, alongside IL-6, TRAIL, IFN-γ, MIG, and M-CSF, showed consistent differential expression between the non/low response group and the moderate strong response group at birth and 12 months of age, suggesting a broader network of cytokines contributing to the post-vaccination immune response. However, there was no related study revealed the roles of IL-5, IL-6, TRAIL, IFN-γ, MIG, and M-CSF in the immune response to the hepatitis B vaccine in infants born to HBsAg-positive mothers.

To further identify cytokines affecting non/low response to hepatitis B immunization, we selected 14 cytokines that were significantly differentially expressed between the two groups at birth for LASSO regression analysis and construction of a ROC clinical prediction model, which identified 4 critical cytokines, respectively, IL-5, HGF, IL-12p40 and β-NGF. Further analysis of the combined ROC curve revealed that IL-5 and HGF could be of high predictive value as predictive cytokines for non/low response, with an AUC of 0.844. Previous studies on the relationship between IL-5 and immune response to hepatitis B vaccine have not been reported. Studies have shown that IL-5 was mainly secreted by Th2 cells (Nakayama et al., 2017). Therefore, the reduced level of IL-5 expression may affect the secretion of Th2-related cytokines and thus decrease the immune response. In addition, IL-5 was a key driver of the Th2 pathway (Gandhi et al., 2016), which could stimulate cell proliferation and differentiation, thus acting as an immune response. There are few studies on the mechanisms of HGF, IL-12p40 and β-NGF in the immune response to hepatitis B vaccine. HGF is a paracrine cell growth factor secreted by mesenchymal cells and targets and acts primarily on epithelial and endothelial cells, but also on hematopoietic progenitor cells and T cells (Ozden et al., 2004). Studies have shown that serum HGF levels in patients with chronic hepatitis B may reflect viral load, necro-inflammatory activity in the liver (Ozden et al., 2004). IL-12 is known as a T cell-stimulating factor and plays a vital role in the activity of natural killer cells and T lymphocytes (Vignali and Kuchroo, 2012). β-NGF is a neurotrophic factor that plays a crucial role in developing and preserving the sensory and sympathetic nervous system (Yuan et al., 2013). In addition, β-NGF also functions as a growth and differentiation factor for B lymphocytes and enhances B cell survival, suggesting that β-NGF may have a potential immunomodulatory role (Hillis et al., 2016). However, further investigations are needed to elucidate the mechanisms of these cytokines in the immune response to hepatitis B vaccine.

The non/low immune response to hepatitis B immunization in infants born to HBsAg-positive mothers may be associated with decreased IL-5 and HGF expression levels and birth weight, and the combined IL-5 and HGF index could effectively predict the vaccine immune response in infants born to HBsAg-positive mothers. The predictive value of IL-5 and HGF on immune response was found, and further studies on whether IL-5 and HGF can be used as vaccine adjuvants and the mechanisms of IL-5 and HGF in the immune response can follow. Indeed, there are some limitations in this study. One limitation of our study is its relatively small sample size, which may not fully represent the broader population of infants born to HBsAg-positive mothers. Additionally, while we have identified significant associations between certain cytokines and the immune response to hepatitis B vaccination, our study is observational and cannot definitively establish causality. Further research involving larger, diverse cohorts and experimental studies are necessary to validate our findings and elucidate the underlying mechanisms of the immune response to hepatitis B vaccination in this specific population.

In summary, the present study is the first to investigate the associations between 48 immune-related cytokines and the immune response to hepatitis B immunization in infants born to HBsAg-positive mothers. Our study identified 2 key cytokines for predicting the immunization response to hepatitis B vaccine for infants born to HBsAg-positive mothers, providing a theoretical basis for developing and implementing effective immunotherapies against non/low immune response in infants in the future clinical practice.

## Data availability statement

The original contributions presented in the study are included in the article/**Supplementary Material**. Further inquiries can be directed to the corresponding author.

## Ethics statement

The studies involving humans were approved by the ethics committees of The Third People’s Hospital of Shenzhen. The studies were conducted in accordance with the local legislation and institutional requirements. The human samples used in this study were acquired from a by- product of routine care or industry. Written informed consent for participation was not required from the participants or the participants’ legal guardians/next of kin in accordance with the national legislation and institutional requirements.

## Author contributions

GO: Data curation, Formal analysis, Methodology, Software, Visualization, Writing – original draft, Writing – review & editing. LQ: Investigation, Data curation, Formal analysis, Methodology, Writing – original draft. LZ: Data curation, Formal analysis, Investigation, Methodology, Funding acquisition, Writing – original draft. YY: Methodology, Writing – review & editing. GY: Methodology, Writing – review & editing. LP: Methodology, Writing – review & editing. YaL: Methodology, Funding acquisition, Writing – review & editing. LY: Investigation, Writing – review & editing. YiL: Conceptualization, Funding acquisition, Project administration, Resources, Supervision, Writing – review & editing.
