# Supplementary material for: Cytokine IL-5 and HGF: combined prediction of non-/low immune response to hepatitis B vaccination at birth in infants born to HBsAg-positive mothers
Source: Front Cell Infect Microbiol. 2024 Mar 1;14:1332666. doi: 10.3389/fcimb.2024.1332666 (PMC10940320; doi:10.3389/fcimb.2024.1332666)
Supplement: Supplementary file 1 [file DataSheet_1.docx]

**Table S1**. The information of selected 48 cytokines in present study.

| Cytokines | Description | Cytokines | Description |
| --- | --- | --- | --- |
| IL-1β | interleukin 1 beta | IFN-γ | Interferon gamma |
| IL-1Ra | interleukin 1 receptor antagonist | GRO-α | Growth regulated oncogene α |
| IL-1α | interleukin 1 alpha | HGF | Hepatocyte growth factor |
| IL-2Ra | interleukin 2 receptor antagonist | IFN-α2 | Interferon alpha-2 |
| IL-2 | interleukin 2 | LIF | Leukemia Inhibitory Factor |
| IL-3 | interleukin 3 | MCP-3 | Monocyte Chemotactic Protein 3 |
| IL-4 | interleukin 4 | IP-10 | IFN-gamma-inducible protein 10 |
| IL-5 | interleukin 5 | MIG | Mitogen-inducible gene |
| IL-6 | interleukin 6 | β-NGF | Beta nerve growth factor |
| IL-7 | interleukin 7 | SDF-1α | Stromal cell-derived factor 1α |
| IL-8 | interleukin 8 | MIP-1α | Macrophage Inflammatory Protein-1 alpha |
| IL-9 | interleukin 9 | MIP-1β | Macrophage Inflammatory Protein-1 beta |
| IL-10 | interleukin 10 | PDGF-BB | Platelet-derived growth factor |
| IL-12 (p70) | interleukin 12 (p70) | TNF-α | tumor necrosis factor alpha |
| IL-12 (p40) | interleukin 12 (p40) | VEGF | Vascular endothelial growth factor |
| IL-13 | interleukin 13 | CTACK | cutaneous T-cell-attracting chemokine |
| IL-15 | interleukin 15 | MIF | Macrophage migration-inhibitory factor |
| IL-16 | interleukin 16 | MCP-1(MCAF) | Monocyte chemoattractant protein-1/monocyte chemotactic and activating factor |
| IL-17 | interleukin 17 | SCGF-β | Stem cell growth factor-beta |
| IL-18 | interleukin 18 | M-CSF | Macrophage colony Stimulating Factor1 |
| FGF basic | basic fibroblast growth factor | TNF-β | Tumor necrosis factor-β |
| Eotaxin | Eotaxin | SCF | Stem cell factor |
| G-CSF | Granulocyte colony stimulating factor | GM-CSF | Granulocyte-macrophage colony stimulating factor |
| TRAIL | TNF-related apoptosis-inducing ligand | RANTES | regulated on activation, normal T cell expressed and secreted |

**Table S2.** Comparison of the general clinical data of HBsAg-positive mothers between the non/low-response and moderate strong response groups.

| Characteristics | non/low-response  (n=13) | moderate strong response  (n=87) | *χ^2^/t/Z* | P value |
| --- | --- | --- | --- | --- |
| Age | 30.0 (27.0, 33.0) | 29.0 (26.0, 32.0) | -0.510 | 0.610 |
| Gestational week | 39.37 ± 1.29 | 39.21 ± 1.07 | 0.174 | 0.622 |
| ALT (U/L) | 15.0 (8.5, 17.5) | 13.0 (9.0, 21.0) | -0.277 | 0.782 |
| AST (U/L) | 18.0 (15.5, 27.5) | 20.8 (16.5, 25.0) | -0.236 | 0.813 |
| TBil (μmol/L) | 6.3 (5.5, 7.9) | 7.7 (5.7, 10.0) | -1.322 | 0.186 |
| ALB (g/L) | 32.5 (28.9, 35.0) | 35.2 (32.3, 36.7) | -1.963 | 0.050 |
| BUN (μmol/L) | 3.7 (3.0, 3.9) | 3.1 (2.6, 3.9) | -1.210 | 0.226 |
| Cr (mmol/L) | 50.0 (43.0, 55.0) | 48.0 (42.0, 55.0) | -0.256 | 0.798 |
| WBC (10^9/L) | 10.89 ± 3.65 | 9.80 ±2.67 | 1.306 | 0.195 |
| HGB (g/L) | 110.15 ± 4.78 | 112.46 ± 13.00 | -1.199 | 0.237 |
| PLT (10^9/L) | 227.85± 68.05 | 214.84 ± 56.89 | -0.749 | 0.455 |
| HBV-DNA (log_10_IU/mL) | 2.1 (1.3, 6.9) | 2.5 (1.3, 4.8) | -0.450 | 0.653 |
| Primiparas, n (%) | 7 (53.8) | 45 (51.7) | 0.020 | 0.886 |
| HBeAg-positive, n (%) | 6 (46.2) | 48 (55.2) | 0.370 | 0.543 |
| Maternal comorbidities | | | | |
| PROM, n (%) | 2 (15.4) | 12 (13.8) | / | 1.000 |
| ICP, n (%) | 1 (7.7) | 4 (4.6) | / | 0.509 |
| GDM, n (%) | 0 (0.0) | 10 (11.5) | / | 0.351 |
| BLD, (ml) | 200 (100, 300) | 200 (140, 300) | -0.016 | 0.988 |

ALT, alanine aminotransferase; AST, aspartate aminotransferase; TBil, total bilirubin; ALB, albumin; BUN, blood urea nitrogen; Cr, creatinine; WBC, white blood cell count; HGB, Hemoglobin; PLT, platelet; PROM, premature rupture of membrane; ICP, Intrahepatic cholestasis of pregnancy; GDM, gestational diabetes mellitus; BLD, Blood loss at delivery.

**Table S3.** Comparison of the general clinical data of infants born to HBsAg-positive mothers between the non/low-response and moderate strong response groups.

| Characteristics | non/low-response  (n=13) | moderate strong response  (n=87) | *χ^2^/t/Z* | P value |
| --- | --- | --- | --- | --- |
| Mode of delivery, n (%) |  |  | 3.191 | 0.074 |
| Natural delivery | 5(38.5) | 56(64.4) |  |  |
| Cesarean delivery | 8(61.5) | 31(35.6) |  |  |
| Gender, n (%) |  |  | 0.774 | 0.379 |
| Male | 9(69.2) | 49(56.3) |  |  |
| Female | 4(30.8) | 38(43.7) |  |  |
| Mode of infant feeding, n (%) |  |  | 6.012 | 0.050 |
| Breastfeeding | 6(46.2) | 33(37.9) |  |  |
| Artificial feeding | 6(46.2) | 20(23.0) |  |  |
| Mixed feeding | 1(7.6) | 34(39.1) |  |  |
| Birth weight (g) | 3600 (3325, 3800) | 3200 (3000, 3500) | -2.840 | 0.005 |

**Table S4**. The parameter of Lasso-Logistic regression with Binomial Deviance.

|  | Lambda | Index | Measure | SE | Nonzero |
| --- | --- | --- | --- | --- | --- |
| Lambda. min | 0.05582 | 78 | 0.7461 | 0.1135 | 4 |
| Lambda. lse | 0.11353 | 1 | 0.7948 | 0.1315 | 0 |

**Table S5**. The coefficients of variables in the LASSO regression model.

| Number | Variables | Coefficients |
| --- | --- | --- |
| 1 | IL-5 | -1.1712E-03 |
| 2 | HGF | -9.2408E-06 |
| 3 | IL-12p40 | -7.9706E-05 |
| 4 | β-NGF | -5.3199E-06 |


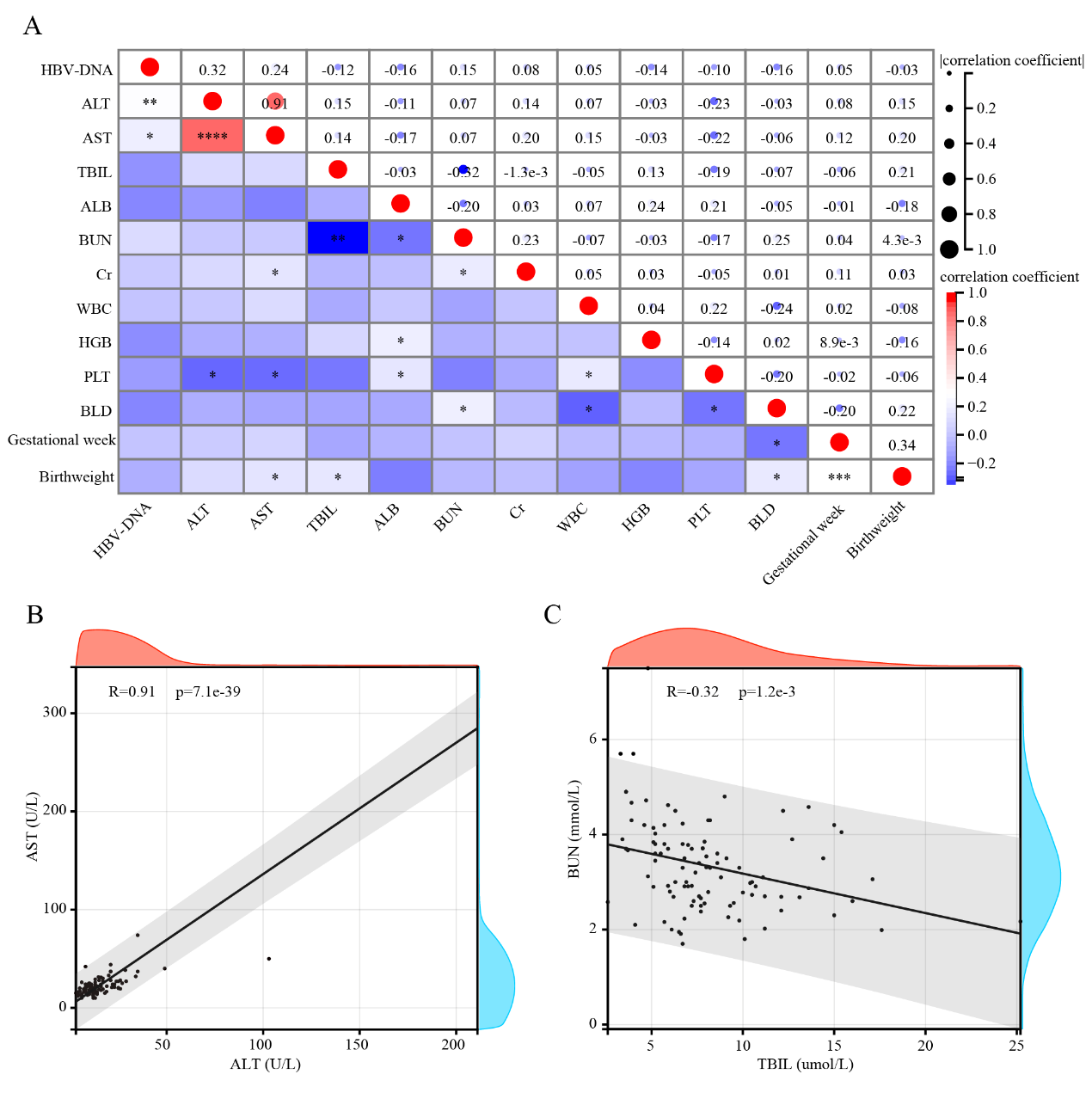


**Figure S1. The clinical correlation between each of the clinical parameters.** (A) The correlation heatmap showed the correlation between each of the clinical parameters of HBsAg-positive mothers. (B)The correlation between ALT and BLT. (C)The correlation between TBIL and BUN. Pearson’s correlation analysis was used to depict the correlation. P<0.05 indicated statistical significance. P-values were showed as: *, P <0.05; **, P <0.01; ***, P <0.001; ****, P <0.0001.


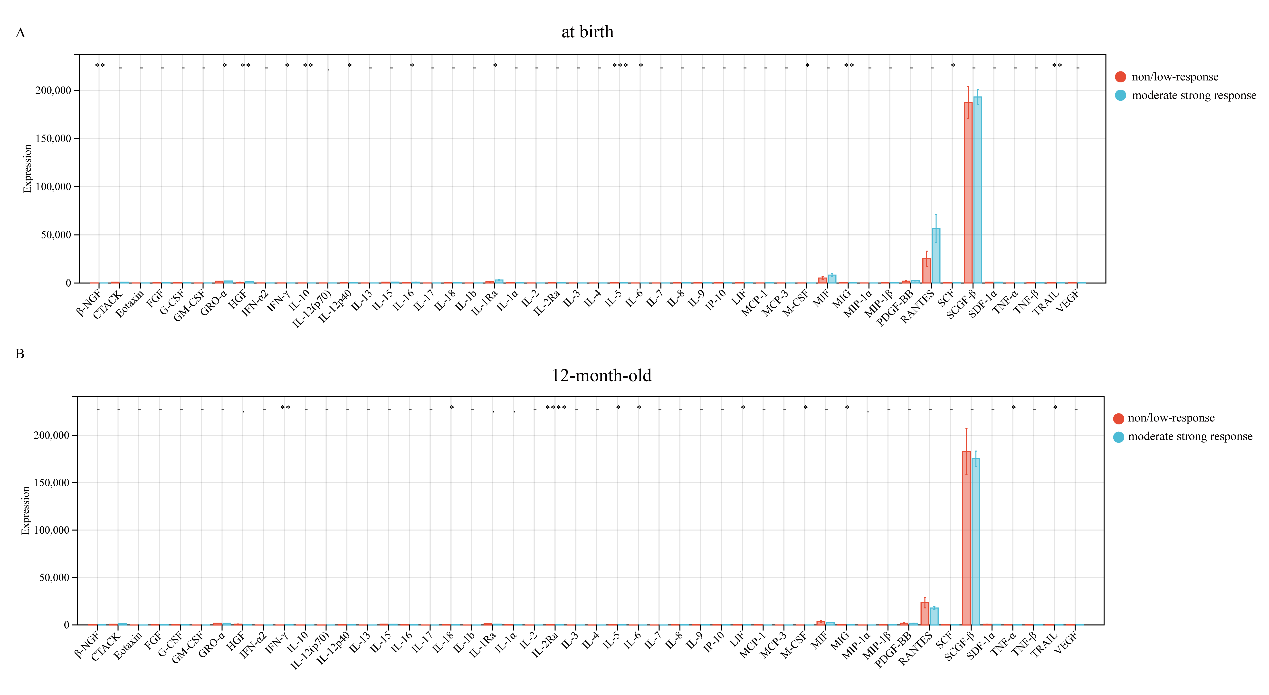


**Figure S2. The differential expression level of 48 cytokines in infants born to HBsAg-positive mothers at birth (A) and at 12 months of age (B).** P<0.05 indicated statistical significance. P-values were showed as: *, P <0.05; **, P <0.01; ***, P <0.001; ****, P <0.0001.
